# A shortened version of Raven’s standard progressive matrices for children and adolescents

**DOI:** 10.1111/bjdp.12381

**Published:** 2021-05-27

**Authors:** Anna M. Langener, Anne‐Wil Kramer, Wouter van den Bos, Hilde M. Huizenga

**Affiliations:** ^1^ Groningen Institute for Evolutionary Life Sciences University of Groningen The Netherlands; ^2^ Department of Developmental Psychology University of Amsterdam The Netherlands; ^3^ Center for Adaptive Rationality, Max Planck Institute for Human Development Berlin Germany; ^4^ Amsterdam Brain and Cognition Center The Netherlands; ^5^ Research Priority Area Yield Amsterdam The Netherlands

**Keywords:** progressive matrices, short form, developmental research, general cognitive ability, penalized regression

## Abstract

Numerous developmental studies assess general cognitive ability, not as the primary variable of interest, but rather as a background variable. Raven’s Progressive Matrices is an easy to administer non‐verbal test that is widely used to measure general cognitive ability. However, the relatively long administration time (up to 45 min) is still a drawback for developmental studies as it often leaves little time to assess the primary variable of interest. Therefore, we used a machine learning approach – regularized regression in combination with cross‐validation – to develop a short 15‐item version. We did so for two age groups, namely 9 to 12 years and 13 to 16 years. The short versions predicted the scores on the standard full 60‐item versions to a very high degree *r* = 0.89 (9–12 years) and *r* = 0.93 (13–16 years). We, therefore, recommend using the short version to measure general cognitive ability as a background variable in developmental studies.

**Statement of contribution**

***What*** ***is already known on this subject?***

- Raven's Standard Progressive Matrices is widely used to measure cognitive ability as background variable in developmental studies.
- A drawback is its long administration time (up to 45 min), and it would therefore be helpful to develop a shortened version.
- Although short versions of the RSPM exist, no short version is suitable for children and adolescents.

***What*** ***does this study add?***

- We used a machine learning approach to develop shortened 15‐item versions for two age groups (9–12 and 13–16 years).
- Results showed that the short versions predicted scores on the original version to a high degree, and would thus be suitable as an alternative to the original version.

## Introduction

In developmental studies, general cognitive ability is often measured as a background variable, rather than a variable of interest (see, for example, Cheung, Chan, & Tsui, 2016; Meinhardt‐Injac, Daum, & Meinhardt, 2020). Hence, a general cognitive ability test is added to the test battery, significantly lengthening the test administration. Especially for children and adolescents, this might be an issue due to a limited attention span and limited time to take part in research. As a result, measuring general cognitive ability may substantially interfere with the measurement of interest. Therefore, it is of importance that a short test is available for children and adolescents. Here, we develop such a test based on the frequently used Raven’s Progressive Matrices (RPM).

The RPM is a non‐verbal test that is widely used to assess ‘general cognitive ability’ (Raven, 1989). Three different versions of the RPM exist, the Raven standard progressive matrices (RSPM), the coloured progressive matrices (CPM), which is easier, and the advanced progressive matrices (APM), which is more difficult. The original 60‐item RSPM is the most frequently used version and can be used for all age groups. Each item shows a geometric pattern with a missing piece. Participants must select the correct answer option that completes the pattern. The test consists of five sets (A, B, C, D, and E) each with 12 items. Each set becomes progressively more difficult. Originally published in 1938, the test has been analysed over the years and is regarded as a valid indicator of general cognitive ability worldwide (Pind, Gunnarsdóttir, & Jóhannesson, 2003; Raven, 2000). Although there are many advantages in using a well‐validated psychometric instrument, one limitation is the long administration time, which is often prohibitive for developmental populations.

Shortened RSPMs have been proposed for adult samples. Elst et al. (2013) evaluated a shortened version with 36 items (set B, C, D) of the RSPM, and Bilker et al. (2012) created a short version of the RSPM with only nine items. Given that both short versions were based on an adult sample, these may not suitable for children and adolescents. For instance, items on the easier sets (A, B) may be more predictive for the final score for children compared with adults (who probably have all of these correct). Similar short versions exist for the CPM and the APM (e.g., Arthur & Day, 1994; Bors & Stokes, 1998; Smits, Smit, van den Heuvel, & Jonker, 1997), but none of these were derived in developmental populations. Finally, there is a timed version of the RPSM, with a 20‐min time limit, validated in an adult sample (Hamel & Schmittmann, 2006). However, a potential drawback of the timed version is that it may have a two‐dimensional structure, that is, ability and intellectual efficiency (as noted by Raven, Raven, & Court, 1993, 1998), instead of a one‐dimensional structure, ability (Hamel & Schmittmann, 2006). In conclusion, no short version of the RSPM currently exists that is suitable for children and adolescents.

In this study, we focused on the RSPM, because it is widely used and often considered to be one of the best measures for cognitive ability (Perret & Dauvier, 2018; Qiu, Hatton, & Hou, 2020). Since the best set of items to assess individual differences in general cognitive ability is likely to be different in different age groups, our goal was to develop separate short versions for two age groups (age 9–12 and 13–16 years). We analysed existing RSPM data for these two age groups separately. We used penalized regression in a cross‐validation set‐up as an item‐reduction technique. That is, we estimated first in one part of the data which subset of items was best predictive of the total Raven score, and we did this using penalized regression, a technique especially suited for prediction purposes (e.g., Matsui & Konishi, 2011; Tibshirani, 1996; Zou & Hastie, 2005). In the second part of the data, we then determined how well this earlier identified subset of items predicted the total Raven score.

## Method

### Sample characteristics

We requested existing data from several research groups. For the younger age group (around 9 to 12 years), we received one data set (*n* = 298) from the Orwell (Oral and written language learning) study of the University of Amsterdam, the Netherlands (Van Koert et al., 2019). The sample characteristics can be found in Table 1. In the Netherlands, 9‐ to 12‐year‐olds go to school together irrespective of their capacities. So, we expect the mean and variance of the general cognitive ability to be representative of the general population.

**Table 1:** Overview data sets

	Data set	*N*	Age			RSPM score		
			*M* (*SD*)	min	max	*M* (*SD*)	min	max
Younger age group	Orwell study	289	N.A. (N.A.)*	N.A.*	N. A.*	37.17 (6.91)	*15*	*56*
Older age group	Data set 1	557	13.14 (0.36)	12.52	14.55	44.43 (6.63)	10	59
	Data set 2	157	13.99 (0.39)	12.61	14.65	44.59 (6.82)	19	56
	Data set 3	273	14.75 (0.72)	13.03	17.3	42.15 (7.45)	11	58
	Total	987	13.72 (0.86)	12.52	17.3	43.83 (6.97)	10	59

Note

*N* is calculated after omitting missing values.

* Due to data protection reasons, we did not receive the exact age for each participant.

For the older age group (around 13 to 16 years), we received three data sets (*n* = 1,071; see Acknowledgements). The sample characteristics are depicted in Table 1. Most participants (82.4%) were from the pre‐(applied)university school level in the Netherlands (HAVO or VWO). The other participants (17.6%) were from pre‐vocational school level in the Netherlands (MAVO). Note that in the third data set, the timed version of the RSPM (Hamel & Schmittmann, 2006) was used, and thus, the data might have a two‐dimensional structure instead of a one‐dimensional structure.

Participants with missing values (younger age group: *n* = 11, older age group: *n* = 43,) and duplicates within the data set (younger age group: *n* = 0, older age group: *n* = 4) were excluded from the analysis. For the older age group, participants who were younger than 12.5 years (*n* = 37) were excluded as well to avoid overlapping ages in the two different age groups. Items A1 and A2 (the first and second item of set A) were used as practice items in the younger age group and were therefore omitted.

### Item selection with penalized regression

We used penalized regression to select a subset of items that could predict the total test score of each participant. This subset of items could then be used instead of the 60‐item RSPM. Penalized regression adds a penalty and tuning parameter to the linear regression model and thereby reduces the coefficients of less essential items to zero (Tibshirani, 1996; Zou & Hastie, 2005). Thus, we obtain a subset of items that predicts the total score on the RSPM best. Penalized regression requires that we choose a penalty parameter (λ). Additionally, we must choose a tuning parameter (α), which determines the relative influence of the so‐called Ridge and Lasso penalty. In the next paragraph, it is explained how these penalty and tuning parameters are chosen. When α is equal to one, only the Lasso penalty is used, and when α equals zero, only the Ridge penalty is used. A value between zero and one means that Elastic Net Penalty is chosen, which thus combines the Lasso and Ridge penalty (Zou & Hastie, 2005). All calculations were done in R with the package Glmnet (Friedman, Hastie, & Tibshirani, 2010).

**Figure 1:**
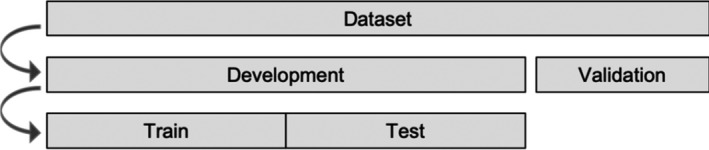
Splitting of the data set.

We split our data set randomly in a development (80%) and validation (20%) set (Figure 1). For the younger age group (9–12), the validation set contained 58 participants and for the older age group (13–16) 199 participants. In order to find the best penalty and tuning parameters (lambda and alpha), we randomly split the development set again in a train set (40%) and test set (40%).

**Figure 2:**
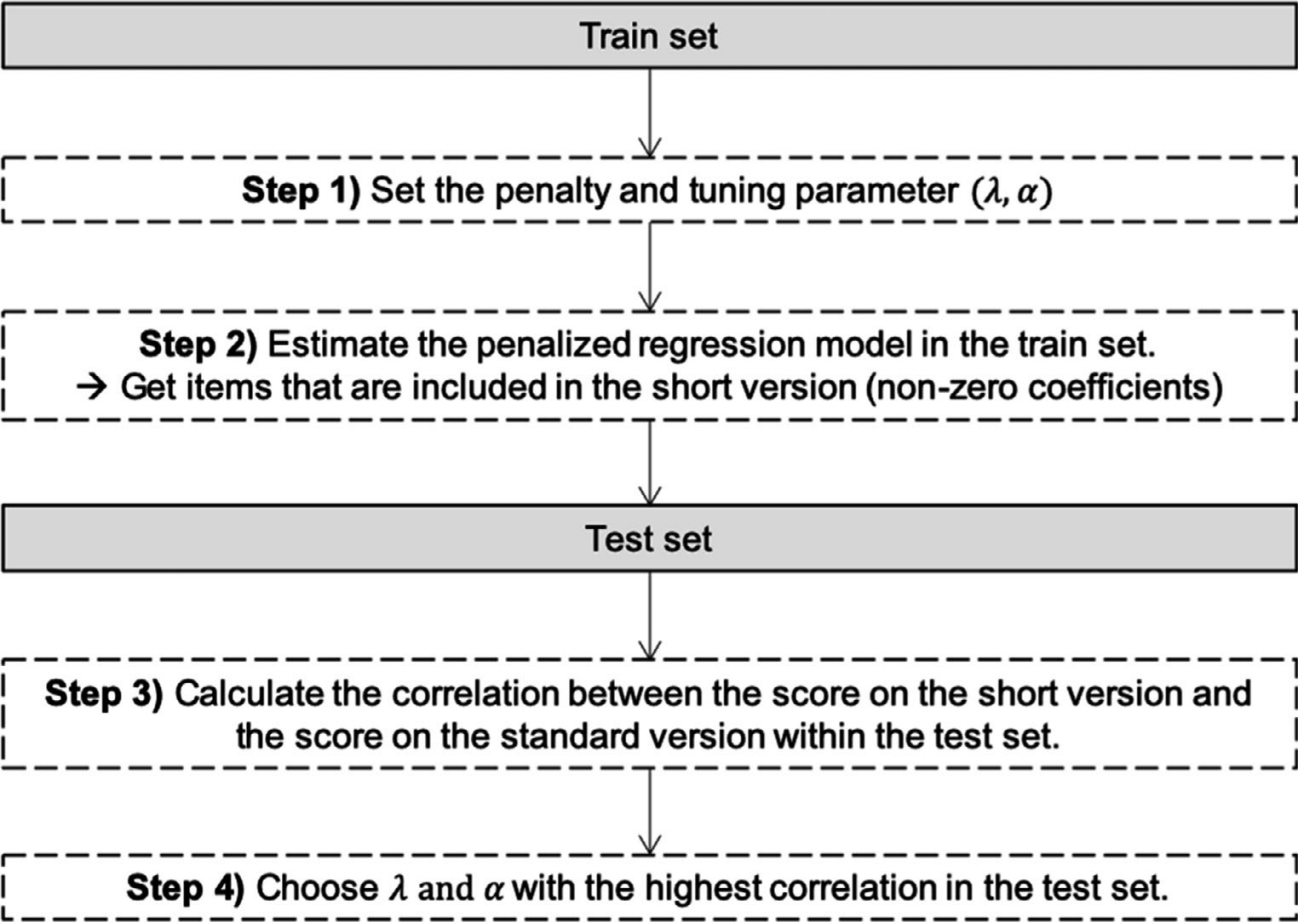
Algorithm to choose the best tuning parameter.

With the aim of finding the best value for the penalty and tuning parameters, we used the algorithm depicted in Figure 2. First, we set an initial value for the penalty and tuning parameters. Second, we estimate the penalized regression model. By doing so, we obtained a coefficient for each item. Some coefficients were shrunk to zero, and thus, these are not included in the initial short version. Third, we examined the correlations between the initial short version and the long version in the test set (cf. Figure 2). We repeated this algorithm for different combinations of penalty and tuning parameters (λ = 2 to 3.5, α = 0.5 to 1). A higher penalty parameter sets the weight of more coefficients to zero. Thus, a higher penalty parameter leads to the selection of fewer items. Therefore, with varying the penalty and tuning parameters we obtain various short versions differing in the number of items that are selected. Short versions with the same length may contain different items; consequently, we chose the penalty and tuning parameters that lead to the short version with the highest correlation in the test set. Due to practical reasons (limited amount of time, limited attention span), we decided that our short version should not exceed the length of 15 items.

### Validation

We examined in the validation set the correlation between the sum score on the final short form and the sum score on the original 60‐item version.

**Figure 3:**
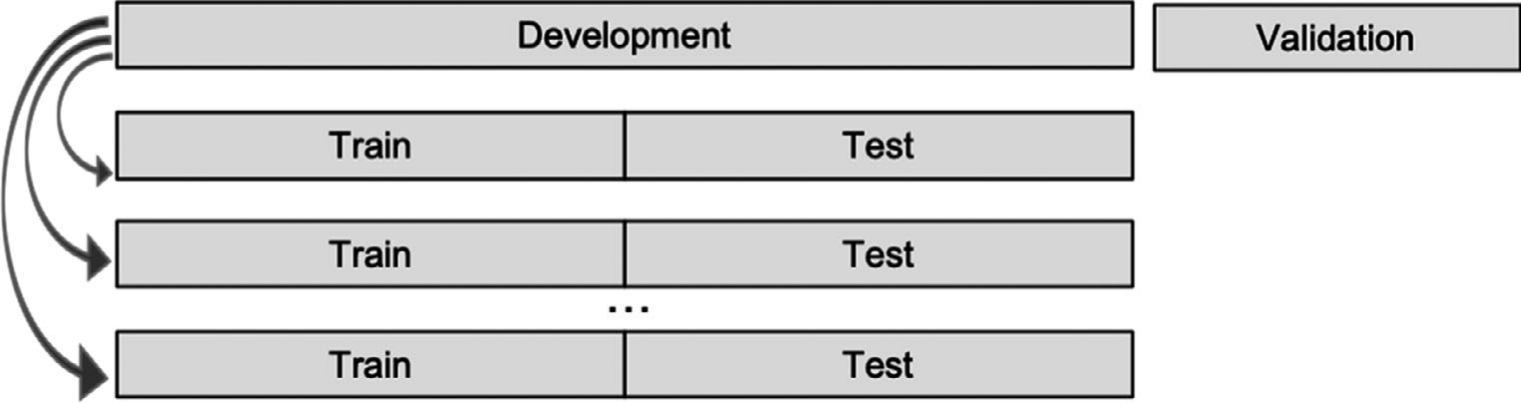
Monte Carlo cross‐validation.

Furthermore, we performed an additional analysis, namely Monte Carlo cross‐validation, in order to check whether our results are robust. That is, we applied the same procedure again as described above just with a different random split of the train and test set (Figure 3). Meaning that we split our development set randomly in a different train and test set for each iteration. Afterwards, we calculated again the best penalty and tuning parameters as described above. We retested the procedure 100 times and examined whether the same items were selected.

Additionally, we checked for floor and ceiling effects. Thus, we checked if our short version measures well for the low and high ability range. Therefore, we examined the density plot of Raven’s score on the short version. A skewed density plot to the left entails that the test might not measure well for participants who score below average. Similarly, a skewed density plot to the right entails that the test does not measure well for participants who score above average, which would indicate a ceiling effect.

Next, we investigated how well our chosen method performs compared with a traditional item‐reduction technique, namely Item Response Theory (IRT; Hambleton & van der Linden, 1982). Please see the Appendix S1 for more information regarding the item selection with IRT.

Finally, we examined how well our developed short form performs compared with 15 randomly selected unique items. To this end, we randomly selected a subset of 15 unique items out of 60 items. Afterwards, we examined the correlation between the obtained random short form and the full form in the validation set. We did this 100 times.

## Results

### Penalized regression

For the first age group, the following fifteen items were selected for the short version: A12, B5, B9, B10, B11, C8, C9, D4, D5, D6, D7, D9, D10, E1, and E5. For the second age group, the following items were selected: A6, B10, B12, C11, D1, D2, D4, D6, D9, D10, E2, E3, E4, E5, and E6. This result indicated that for the youngest age group, more easy items were included as compared with the oldest age group.

### Validation

We randomly split our data set in a development and validation data set. The latter was not involved in the test construction process. For the youngest age group, the correlation between the sum score on the short version and the sum score on the original 60‐item version was *r* = .89. For the oldest age group, the correlation between the sum score on the short version and the original 60‐item version was *r* = .93.

Table 2 shows the validation set correlations between the score on the short version and the score on the original 60‐item version, as a function of a different number of items (length). We required that the short version should not exceed 15 items. Indeed, Table 2 shows that including more items does not add much information.

**Table 2:** Overview of different lengths of the short version

Age group	Lambda	Alpha	Correlation (validation)	Length
Younger age group	3.15	0.55	0.93	20
	2.45	0.7	0.92	19
	3.4	0.55	0.91	18
	2.35	0.8	0.90	17
	3.4	0.6	0.90	16
	3.15	0.65	0.89	15
	2.95	0.7	0.89	14
	2.15	0.95	0.88	13
	3.45	0.65	0.84	12
	2.8	0.8	0.83	11
Older age group	3.3	0.55	0.95	20
	2.15	0.85	0.95	19
	2	0.9	0.95	18
	2.85	0.7	0.94	17
	2.35	0.85	0.93	16
	2.2	0.9	0.93	15
	3.35	0.65	0.91	13
	2.9	0.75	0.91	12
	3.4	0.7	0.90	11

**Figure 4:**
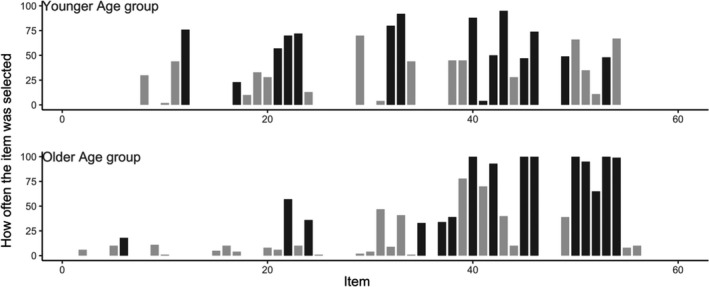
Results Monte Carlo cross‐validation. Items that are used for the short version are depicted in black.

To further investigate the robustness of our selected set of items, we used Monte Carlo cross‐validation. As can be seen in Figure 4, most of the items were selected at least 50% of the cases. Only item D5 was selected in just 4% of the cases. Thus, although there is some variability, indicating other short versions may perform equally well, the set of selected items for the short version is relatively consistent. Figure 4 shows how often an item is included in the short version out of 100 times.

**Figure 5:**
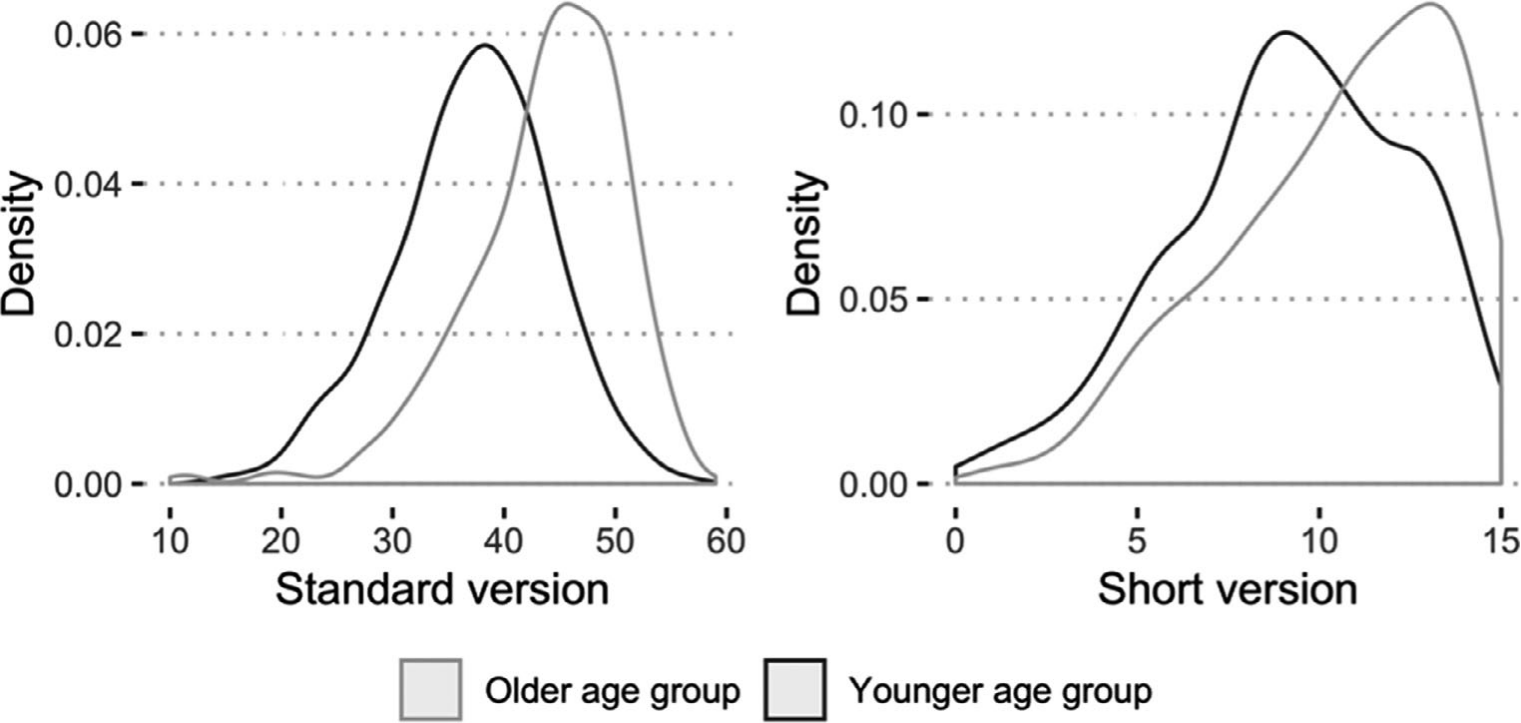
Density of the total Raven’s score on the standard (left) and short (right) version.

To inspect potential ceiling effects, Figure 5 shows the density plot of Raven’s score on the short version and on the standard version. The score of the standard version is skewed to the right, especially for the older age group. This indicates that many individuals answered many items correctly. Our short form inherits this effect for the older age group. Thus, it is likely that a ceiling effect appears in the older age group irrespective of whether the short or long version is used.

Further, we compared the short version obtained by penalized regression with the short version obtained by IRT (see Appendix S1). In the validation set, the short form obtained by Item Response has a slightly lower correlation with the original 60‐item version (youngest age group *r* = .88; older age group *r* = .87 for IRT whereas they were .89 and .93 for penalized regression). Moreover, the short version obtained by IRT has a lower Cronbach’s alpha than the short version obtained by penalized regression (youngest age group α = .65; older age group α = .73 for IRT whereas they were .78 and .80 for penalized regression), which indicates that the short version selected by penalized regression has a higher internal consistency.

**Figure 6:**
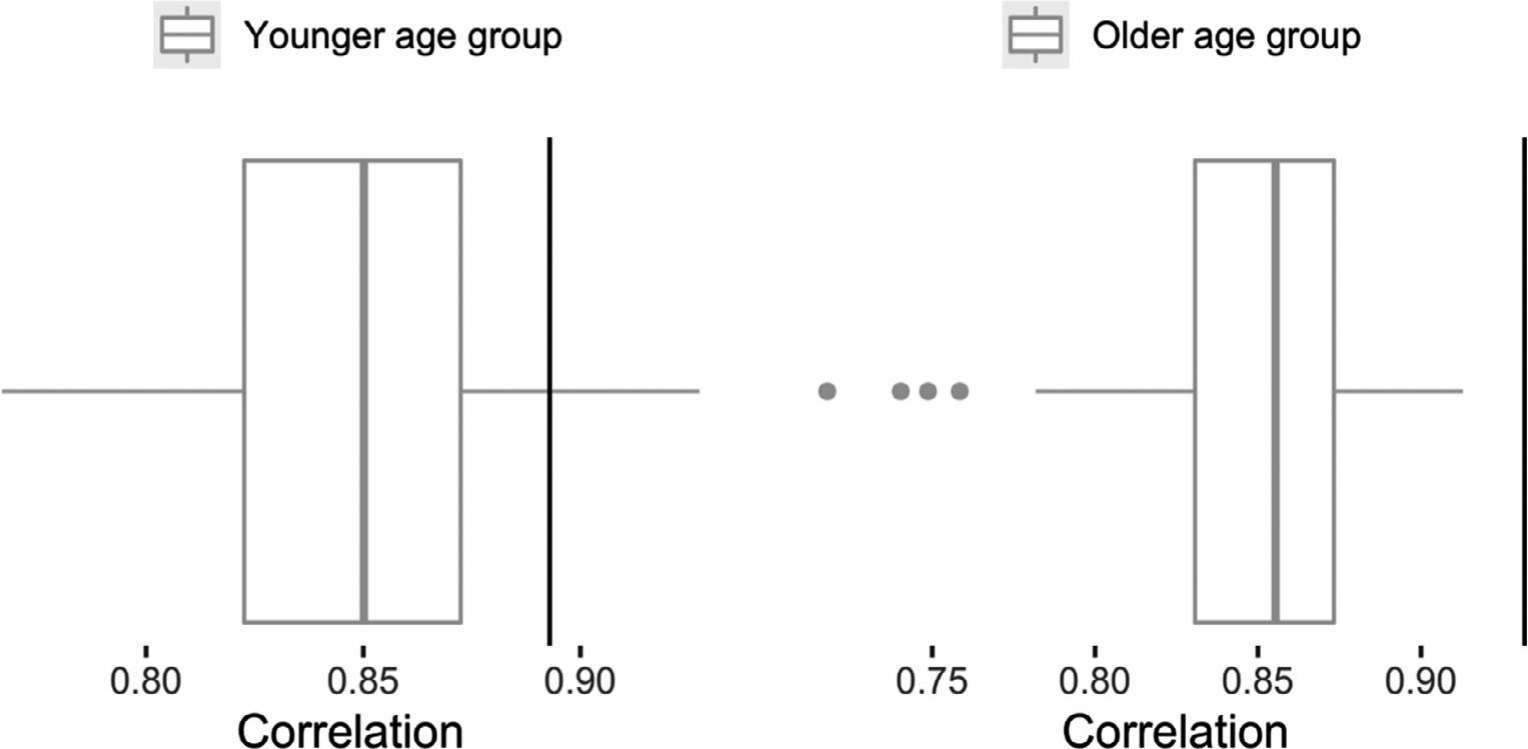
Boxplot of the correlations between the short version and original version for randomly selected subsets of items. The black vertical line indicates the same correlation for the short form obtained by penalized regression.

Finally, we compared the performance of our developed short form with the performance of a randomly selected subset of 15 items. We did this 100 times. For the younger age group, the mean correlation of the randomly selected subsets is *r* = 0.85 (min = 0.77, max = 0.93). Here, our selected subset with penalized regression (*r* = .89) outperforms 94% of the cases. For the older age group, the mean correlation of the randomly selected subsets is *r* = .85 (min = 0.72, max = 0.91). Our selected subset with penalized regression (*r* = .93) outperforms all of the randomly selected subsets. Figure 6 shows the distribution of the 100 correlations compared with the short form obtained by penalized regression. This indicates that our selected short forms perform better than a randomly selected subset of items.

## Discussion

We developed two short versions of the RSPM that showed high predictive validity of the score on the original 60‐item version (*r*’s > .89). These versions consisted of 15 instead of 60 items which reduce the administration time of the RSPM. Therefore, these short versions can confidently be used by developmental researchers that are interested in having a short but reliable proxy for general cognitive ability as a background variable.

We validated our short version with a cross‐validation approach. Most of the items were selected in at least 50% of the cases. However, some items are quite similar; thus, it might be that some of them are easily interchangeable. Bilker et al. (2012) developed a 9‐item short version for an adult sample and similarly concluded that there might be more than one optimal short form. Thus, it seems that the item selection does not depend on the random split of train and test set; however, it could be possible that there are more item combinations that suffice as a short version of the RSPM.

We proposed penalized regression in a cross‐validation set‐up as an item‐reduction technique. To evaluate whether this is an appropriate method, we compared this with another well‐known IRT item‐reduction approach. Indeed, we found that the items selected by penalized regression showed higher predictive values than items selected by IRT.

Several potential limitations warrant discussion. One limitation of our study is that the majority of the sample (86%) from the older age group (13–16) was a combination of above‐average‐ and pre‐university‐level students which means that the items selected might be less representative for average‐level students. For future research, it might be interesting to include individuals from other school levels, mainly students from pre‐vocational schools.

Second, the original 60‐item version of the RSPM shows ceiling effects. Pind et al. (2003) investigated school norms and reported ceiling effects in Iceland’s higher school classes (pupils aged around 13 to 16 years). They concluded that the test is only appropriate for children in the first seven grades (pupils age around 6 to 13 years). Raven (2000) reported ceiling effects as well among young adults. We should be aware that our short version for the older age group most likely inherits this ceiling effect. Thus, for future research, in order to be able to discriminate between general cognitive ability in an adolescent above‐average sample, we recommend to also develop a short version for the Raven's Advanced Progressive Matrices.

Third, due to the smaller number of items, participants are not able to practice with easy items before they get to the more difficult items in the short versions. This may lower their score on the short version. On the other hand, this may also lead to less fatigue and/or boredom (Gonthier & Roulin, 2019). In future research, it may be worthwhile to check whether performance on long and short versions differs.

Last, we developed a test that can be used as a paper–pencil test to assess cognitive ability in adolescents. This has the advantage that the test is easy to use but might miss out on opportunities that require test administration on a computer. In future research, it may be beneficial to develop a short form based on computer adaptive testing. Computer adaptive testing dynamically selects the next item based on the participant's answer on the previous item. Makransky, Dale, Havmose, and Bleses (2016) used computer adaptive testing to develop a short form of the MacArthur–Bates Communicative Development Inventory and found strong evidence that this can be a helpful tool to reduce the test length.

To conclude, we developed two short versions of the RSPM. We did so for children and adolescents separately. The short versions, consisting of 15 instead of 60 items, predict the score on the full‐length test to a high degree, and can therefore be used in developmental research where general cognitive ability is assessed as a background variable.

## Conflicts of interest

All authors declare no conflict of interest.

## Author contribution

Anna Maria Langener, M. Sc. Economics, M. Sc. Psychology (research) (Data curation; Formal analysis; Methodology; Software; Visualization; Writing – original draft; Writing – review & editing) Anne‐Wil Kramer (Conceptualization; Data curation; Investigation; Project administration; Resources; Supervision; Validation; Writing – review & editing) Wouter van den Bos (Conceptualization; Resources; Supervision; Validation; Writing – review & editing) Hilde Huizenga (Conceptualization; Funding acquisition; Supervision; Validation; Writing – review & editing).

## Supporting information


**Appendix S1** Item selection with Item Response Theory.

## Data Availability

Data sharing is not applicable to this article as no new data were created in this study.
