# Supplementary material for: A shortened version of Raven’s standard progressive matrices for children and adolescents
Source: Br J Dev Psychol. 2021 May 27;40(1):35–45. doi: 10.1111/bjdp.12381 (PMC9290746; doi:10.1111/bjdp.12381)
Supplement: Supplementary file 1 — Appendix S1 Item selection with Item Response Theory. [file BJDP-40-35-s001.docx]

**Supplementary Material: A short version of the RSPM for children and adolescents**

**Item selection with Item Response Theory**

Item Response Theory is based on the idea that the probability of success of an item can be predicted by the characteristics of an item and person (Hambleton & van der Linden, 1982). Three commonly used models for a dichotomous response scheme are the one-, two- and the three-parameter logistic model (Hambleton & Swaminathan, 1985, p. 35). The one-parameter model only contains the item difficulty parameter, which indicates how many participants answer the item correctly (Fan, 1998). The two-parameter model contains additionally an item discrimination parameter, which describes how well an item can differentiate between participants. The three-parameter model contains a ‘guessing’ parameter which shows the probability to answer the item correctly just by chance (Fan, 1998).

We decided to use the two-parameter model instead of the three-parameter model as parameters of the three-parameter method are difficult to estimate (Hulme-Lowe, 2016). We decided to use the two- instead of one-parameter model as items differ in their ability of discrimination, meaning that some items can better differentiate between certain ability levels of individuals than other items. Due to this, the two-parameter model was our method of choice.

Following the steps of other researchers (e.g. Edelen & Reeve, 2007; Maples et al., 2015), items were selected based on their discrimination value. We selected those fifteen items that had the highest discrimination value.

**Results Item Response Theory**

The fifteen items with the highest discrimination value for the younger age group are: A5, A6, A8, A9, B2, B3, B5, B9, B10, D1, D2, D4, D5, D7, E6; and for the older age group: A2, A5, A6, A9, A10, B3, B4, D1, D4, D5, D9, D10, E2, E5, E6. The correlation between the score on these items and the total score is *r* = 0.88 for the younger ager group and *r* = 0.87 for the older age group

**Validation: Cronbach’s Alpha**

We evaluated Cronbach’s Alpha and the measurement error to compare our different short versions (Table 1). Cronbach’s Alpha is a measure for the internal consistency of a test. It indicates whether all items belong to the same concept and gives an estimate for the reliability of a test (Cronbach, 1951; Tavakol & Dennick, 2011). The measurement error provides the error variance within the test score (Kline, 1994). Cronbach’s Alpha indicates for all versions a good internal consistency. Only the short version selected by IRT for the younger age group has an acceptable internal consistency. We can see that the Cronbach’s alpha of the short version selected by penalized regression is higher than the short version selected by IRT.

*Table 1.*

*Cronbach’s Alpha.*

|  |  | Cronbach’s Alpha | Measurement error* |
| --- | --- | --- | --- |
| Younger age group  (Validation set, n = 59) | Full version | 0.86 | 0.25 |
|  | Short version penalized regression | 0.77 | 0.41 |
|  | Short version IRT | 0.65 | 0.58 |
| Older age group  (Validation set, n = 197) | Full version | 0.87 | 0.25 |
|  | Short version penalized regression | 0.80 | 0.36 |
|  | Short Version IRT | 0.73 | 0.46 |

*Measurement error is calculated by squaring the Cronbach’s Alpha and subtracting it from one (Kline, 1994).

**Test Standard Error of the Ability Estimate**

We examined IRT based test standard errors to investigate how well our short versions perform for children with different ability levels. The test standard error was calculated based on the two parameter IRT model, with the mirt package (Chalmers, 2012). The test standard errors for the younger age group (see Figure 1) and older age group (see Figure 2) are calculated in the complete dataset, so we did not split it into a leaning and validation set. When calculating the test standard errors for the full version for the younger age group, we removed item A1 and A2 because they were used as test items. We also removed item B2 because every child answered this item correctly.

Self-evidently standard errors are lower in the full version than in the shortened versions, as the full version contains more information. In both age groups, the test standard error for children with low ability levels is larger in our selected short form with penalized regression than in the short form based on IRT. However, our selected short form with penalized regression outperforms the short form based on IRT for medium and high ability levels.

**
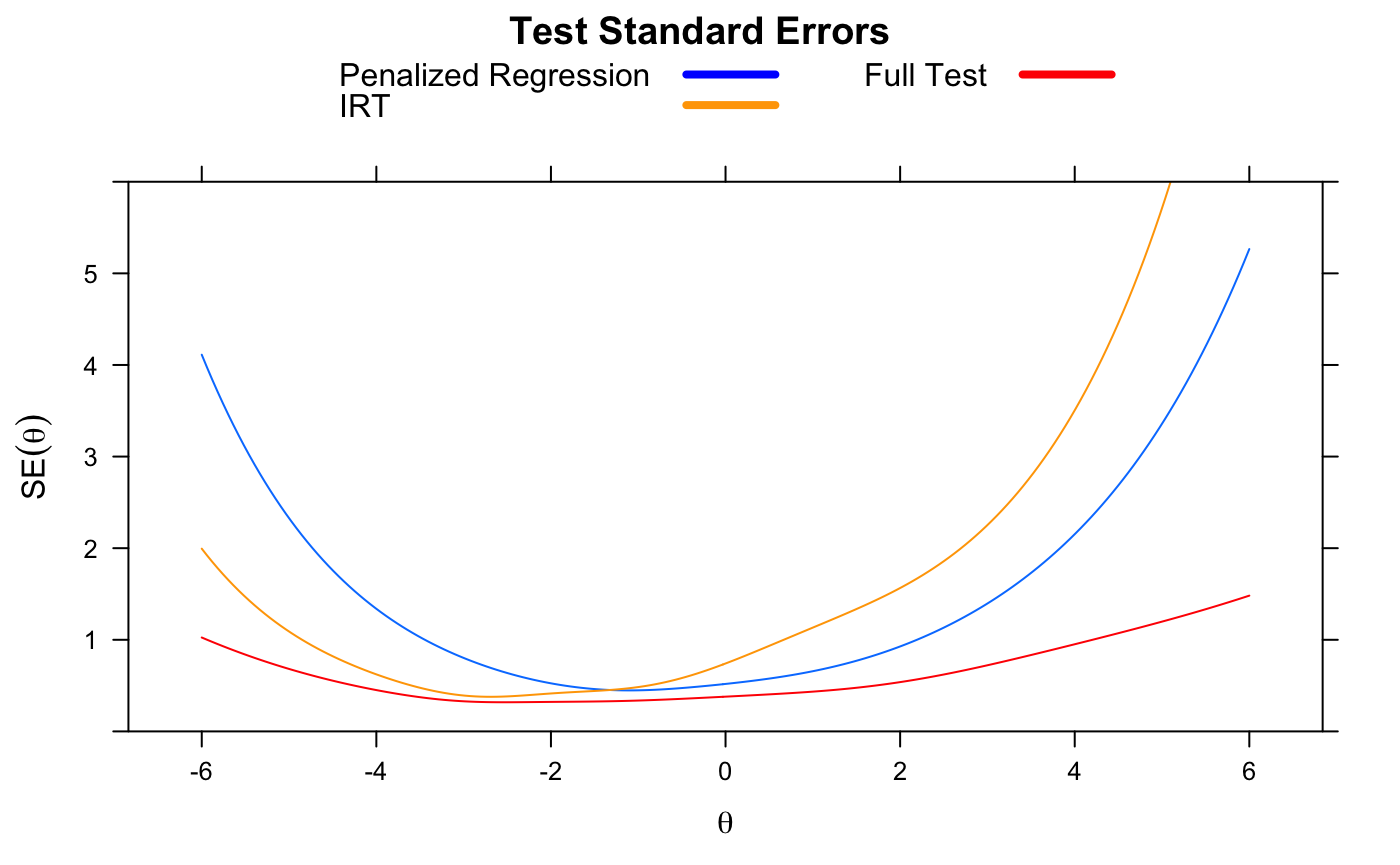
**

*Figure 1.* Test Standard Errors Younger Age Group

**
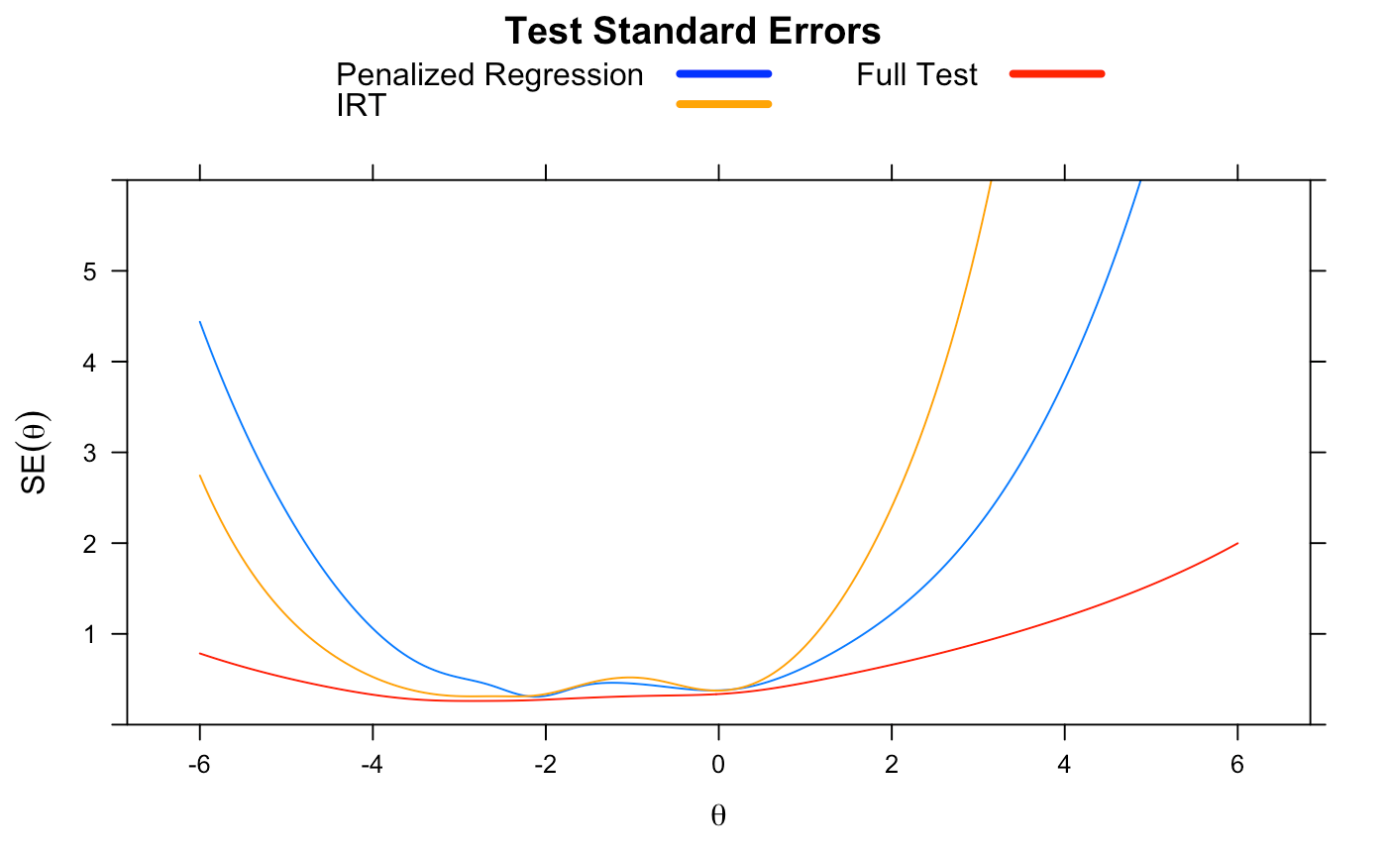
**

*Figure 2.* Test Standard Errors Older Age Group

**References**

Cronbach, L. J. (1951). Coefficient alpha and the internal structure of tests. Psychometrika, 16(3), 297–334.

Chalmers RP (2012). “mirt: A Multidimensional Item Response Theory Package for the R Environment.” Journal of Statistical Software, 48(6), 1–29. doi: [10.18637/jss.v048.i06](https://doi.org/10.18637/jss.v048.i06).

Edelen, M. O., & Reeve, B. B. (2007). Applying item response theory (IRT) modeling to questionnaire development, evaluation, and refinement. Quality of Life Research: an In- ternational Journal of Quality of Life Aspects of Treatment, Care and Rehabilitation, 16 Suppl 1, 5–18. https://doi.org/10.1007/s11136-007-9198-0

Fan, X. (1998). Item Response Theory and Classical Test Theory: An Empirical Comparison of their Item/Person Statistics. Educational and Psychological Measurement, 58, 357– 381. https://doi.org/10.1177/0013164498058003001

Hambleton, R. K., & Swaminathan, H. (1985). Item Response Theory: Principles and Appli- cations. Dordrecht, Springer Netherlands. http://dx.doi.org/10.1007/978-94-017-1988-9

Hambleton, R. K., & van der Linden, W. J. (1982). Advances in Item Response Theory and Applications: An Introduction. Applied Psychological Measurement, 6, 373–378. https://doi.org/10.1177/014662168200600401

Hulme-Lowe, C. (2016). Regularized Marginal Maximum Likelihood: The Use of Shrinkage and Selection Operators for Item Parameter Estimation in the Two-Parameter Logistic Model. Retrieved from the University of Minnesota Digital Conservancy. http://hdl.han dle.net/11299/181741.

Kline, P. (1994). An easy guide to factor analysis. Routledge.

Maples, J. L., Carter, N. T., Few, L. R., Crego, C., Gore, W. L., Samuel, D. B., & Miller, J. D. (2015). Testing whether the DSM-5 personality disorder trait model can be measured with a reduced set of items: An item response theory investigation of the Personality In- ventory for DSM-5. Psychological Assessment, 27, 1195–1210. https://doi.org/10.1037/pas0000120

Tavakol, M., & Dennick, R. (2011). Making sense of Cronbach’s alpha. *International Journal of Medical Education*, *2*, 53–55. https://doi.org/10.5116/ijme.4dfb.8dfd
